# A Mobile App–Based Intervention for Depression: End-User and Expert Usability Testing Study

**DOI:** 10.2196/mental.9445

**Published:** 2018-08-23

**Authors:** Matthew Fuller-Tyszkiewicz, Ben Richardson, Britt Klein, Helen Skouteris, Helen Christensen, David Austin, David Castle, Cathrine Mihalopoulos, Renee O'Donnell, Lilani Arulkadacham, Adrian Shatte, Anna Ware

**Affiliations:** ^1^ School of Psychology Deakin University Geelong Australia; ^2^ Cairnmillar Institute Melbourne Australia; ^3^ School of Science, Engineering & Information Technology Federation University Ballarat Australia; ^4^ School of Psychology Deakin University Melbourne Australia; ^5^ School of Psychological Sciences Monash University Melbourne Australia; ^6^ Black Dog Institute University of New South Wales Sydney Australia; ^7^ Department of Psychiatry University of Melbourne Melbourne Australia; ^8^ School of Health and Social Development Deakin University Geelong Australia

**Keywords:** depression, eHealth, mHealth, young adult

## Abstract

**Background:**

Despite the growing number of mental health apps available for smartphones, the perceived usability of these apps from the perspectives of end users or health care experts has rarely been reported. This information is vital, particularly for self-guided mHealth interventions, as perceptions of navigability and quality of content are likely to impact participant engagement and treatment compliance.

**Objective:**

The aim of this study was to conduct a usability evaluation of a personalized, self-guided, app-based intervention for depression.

**Methods:**

Participants were administered the System Usability Scale and open-ended questions as part of a semistructured interview. There were 15 participants equally divided into 3 groups: (1) individuals with clinical depression who were the target audience for the app, (2) mental health professionals, and (3) researchers who specialize in the area of eHealth interventions and/or depression research.

**Results:**

The end-user group rated the app highly, both in quantitative and qualitative assessments. The 2 expert groups highlighted the self-monitoring features and range of established psychological treatment options (such as behavioral activation and cognitive restructuring) but had concerns that the amount and layout of content may be difficult for end users to navigate in a self-directed fashion. The end-user data did not confirm these concerns.

**Conclusions:**

Encouraging participant engagement via self-monitoring and feedback, as well as personalized messaging, may be a viable way to maintain participation in self-guided interventions. Further evaluation is necessary to determine whether levels of engagement with these features enhance treatment effects.

## Introduction

### Background

Mental illness is both common and costly [1]. People with mental illness have a lower quality of life and poorer work opportunities, and are more likely to attempt suicide [2,3]. Given that onset of depression most often occurs during adolescence to early adulthood [4,5] and that proven treatments for depression become less effective as the severity and duration of the illness increase [6], early detection and treatment of depressive symptoms are seen as key strategies to reduce the prevalence, duration, and burden of depression. Unfortunately, many adolescents and young adults are reluctant to seek help for their depressive symptoms [7], citing barriers such as cost, access to help, lack of anonymity, and perceived stigma of mental illness [8,9]. eHealth technology may help to overcome these barriers to treatment and ultimately assist sufferers in alleviating their depressive symptoms.

### eHealth Interventions for Depression

The past decade has seen increasing use of technology to enhance access to, and engagement with, treatments previously established as efficacious when delivered face-to-face [10,11]. This widespread promotion of eHealth solutions to intervention delivery is premised on the notions that eHealth interventions (1) can replicate treatment gains observed in face-to-face treatment; (2) are acceptable to end users; (3) may have advantages in terms of reach, anonymity, and cost; and (4) provide useful features that may enhance treatment experience and outcomes, such as feedback functions to enable charting of one’s progress, and sophisticated algorithms for tailoring the intervention experience [12,13].

Accumulated evidence broadly supports these premises. Most adolescents and young adults own a smartphone [14], have accessed a mHealth app [15], and report a stated preference for engaging health services in this manner [16]. Importantly, recent meta-analyses suggest that psychological intervention content delivered via a Web- or mobile app can be as efficacious as face-to-face treatment for depression [10,17,18].

Despite these encouraging findings, it is also clear that dropout rates tend to be higher for eHealth interventions than for face-to-face therapy, especially when eHealth interventions are self-guided [19]. The greater dropout rate in self-guided eHealth interventions may signal that the apps are not sufficiently engaging and/or user-friendly to maintain participant interest over time. Check-ins with a clinician or researcher may enable evaluation of whether the content is being used appropriately, whether it is having the desired effect, and whether there is a need to modify the treatment plan. In instances where clinician contact is not feasible, incorporation of persuasive design principles into app development may enhance end-user experience and outcomes for self-guided apps.

### Design Principles to Facilitate Target Behavior

According to the Fogg Behavior Model [20], engagement in a task is dependent on 3 key factors: (1) motivation, (2) sufficient ability for task performance, and (3) triggering to perform the task. This model further stipulates that all the 3 factors are necessary to enable behavior. For instance, motivation alone may be insufficient to bring about change in an individual who does not possess the necessary skills to achieve the desired goal (eg, coping strategies to deal with negative mood). Similarly, triggering performance—whether through an external or internal prompt—may not lead to the desired outcome when the individual is insufficiently motivated to engage in the task at that time.

Within the context of eHealth interventions, incorporation of design features such as engaging, actionable, and easy-to-use content may ensure that ability and motivation levels are less of a barrier to use. Furthermore, personalization of the app through tailoring of content, scheduling of information, and provision of feedback to help participants chart their progress may provide timely prompts that train individuals when to use the content for optimal results. Such features may increase ability and enhance user experience and engagement, and in turn reduce the likelihood of dropout in self-guided treatments [21].

Ultimately, the success of these design choices is determined through usability testing. Jake-Schoffman et al [22] emphasize that usability may be evaluated across a range of dimensions, including how easy the app is to operate, understand, or learn; satisfaction with the app; attractiveness of the layout; and error rates compared with intended usage. Depending on the dimensions one wishes to study, usability may be evaluated using laboratory-based testing [23,24], field testing [25], and/or user feedback [26-28]; however, self-reported usability (hereafter, labeled *perceived usability*) is the most commonly employed usability approach to date for eHealth apps [29].

### The Study

This study sought to evaluate end-user experience of a mobile app-based intervention for depression (BlueWatch) developed with feedback functionality as a central feature to enhance usability and engagement. The app was designed to be self-paced and without therapist input and was targeted at young adults (18 to 25 years), given this is a peak period for the onset of depressive symptoms. Although BlueWatch shares many design features with other commonly available depression treatment apps (evidence-based content, survey, mood monitoring features, etc), a key differentiator is that it uses the participants’ mood survey data to provide real-time messages to help participants work out when best to engage the treatment content.

A mixed methods design was employed to augment quantitative ratings of perceived usability of BlueWatch with qualitative interviewing to flesh out responses to these questions. These questions probed the ease of use and navigability, aesthetic features of the app, integration and suitability of content provided, and app personalization.

The qualities that make an app attractive for users may differ from features that researchers focus on when developing an intervention or the features that clinicians look for in recommending apps to patients. Hence, the second aim of this study was to compare qualitative responses of the user group with app feedback from clinicians with expertise in the treatment of depression and researchers who specialize in mental health intervention research (including a focus on eHealth interventions).

## Methods

### Participants

A total of 5 participants, each from 3 different target groups, were recruited. The majority of participants were female (4 out of 5 for the user and mental health professional groups and 3 out of 5 for the researcher group). The user group had a lower average age (mean 22.4, SD 2.71) than the mental health professional (mean 31.8, SD 6.61) and researcher groups (mean 33.4, SD 5.03).

The target groups included users who had completed use of BlueWatch as part of a randomized controlled trial for individuals with depression (ACTRN12615001093572), mental health clinicians, and mental health researchers. Diagnosis of depression among participants from the user group was ascertained by prescreening with the Patient Health Questionnaire 9 [30], followed by confirmation of diagnosis using the Mini-International Neuropsychiatric Interview [31].

Formative usability trials have demonstrated that a sample of 5 participants can identify 80% of usability issues [32,33], and thus, this study was suitably powered to identify user issues for BlueWatch, both within and across the 3 groups tested. Furthermore, in this study, saturation was reached in the themes derived from qualitative interviews.

### Materials

#### BlueWatch Mobile App Intervention

BlueWatch, a mobile app that comprises short audio activities, journaling exercises, and self-monitoring functions, was designed to improve the well-being and resilience of adults experiencing depressive symptomatology. The app was developed by a multidisciplinary team comprising psychologists, a psychiatrist, and researchers with expertise in eHealth delivery of interventions for depression.

**Figure 1:**
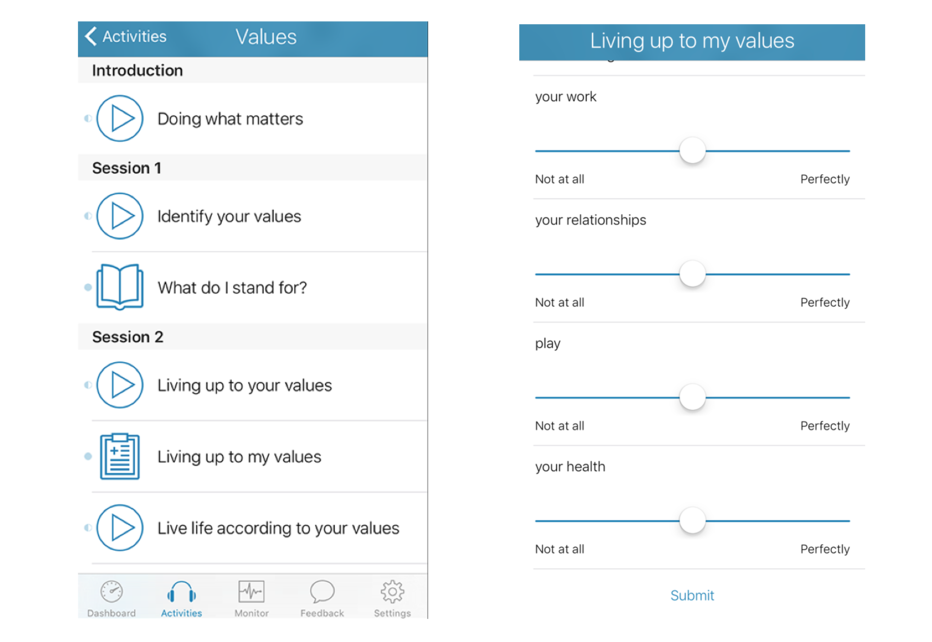
Content for the values module of BlueWatch.

The app was organized into 6 modules based on the principles of cognitive behavioral therapy (CBT): (1) psychoeducation about depression and introduction to CBT, (2) behavioral activation, (3) cognitive restructuring, (4) problem solving, (5) assertiveness skills, and (6) relapse prevention. Each module is based on empirically validated treatment methods and consists of approximately 30 min of content, including instructive text, audio, and various activities to consolidate techniques learned. An example of the content for the values module is provided in Figure 1 to illustrate.

**Figure 2:**
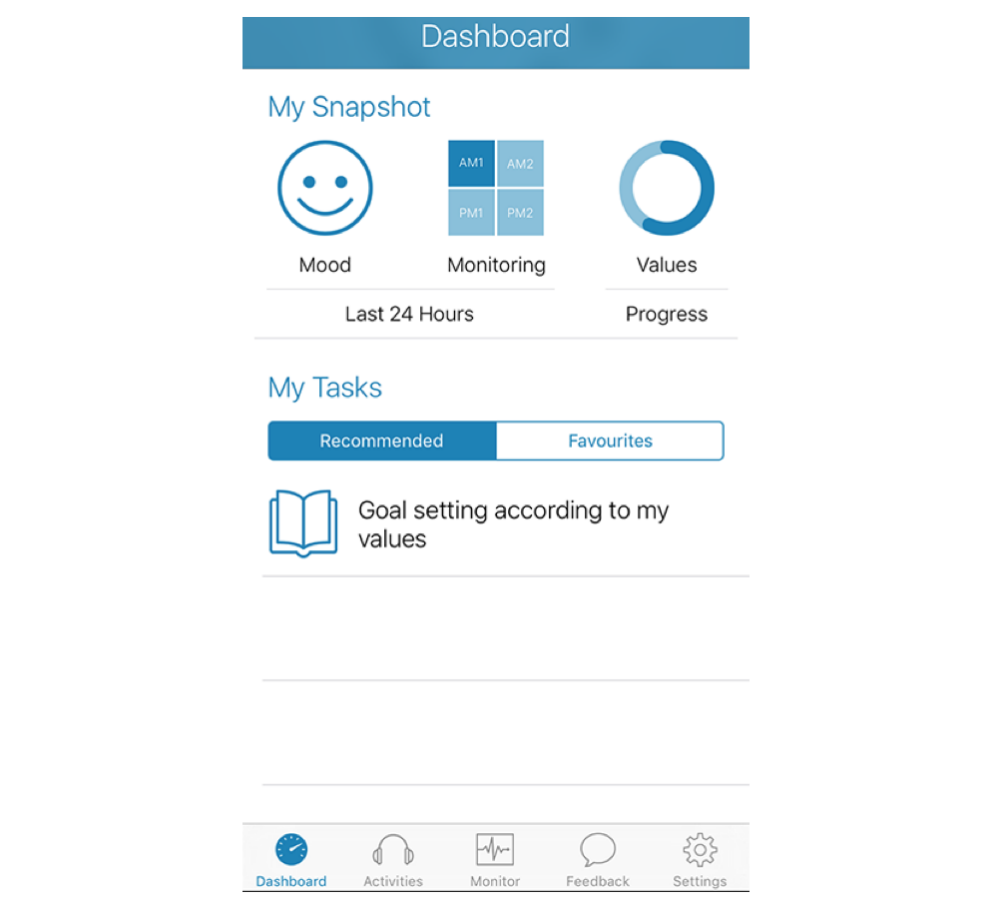
Example of the dashboard used to orient participants to upcoming activities.

**Figure 3:**
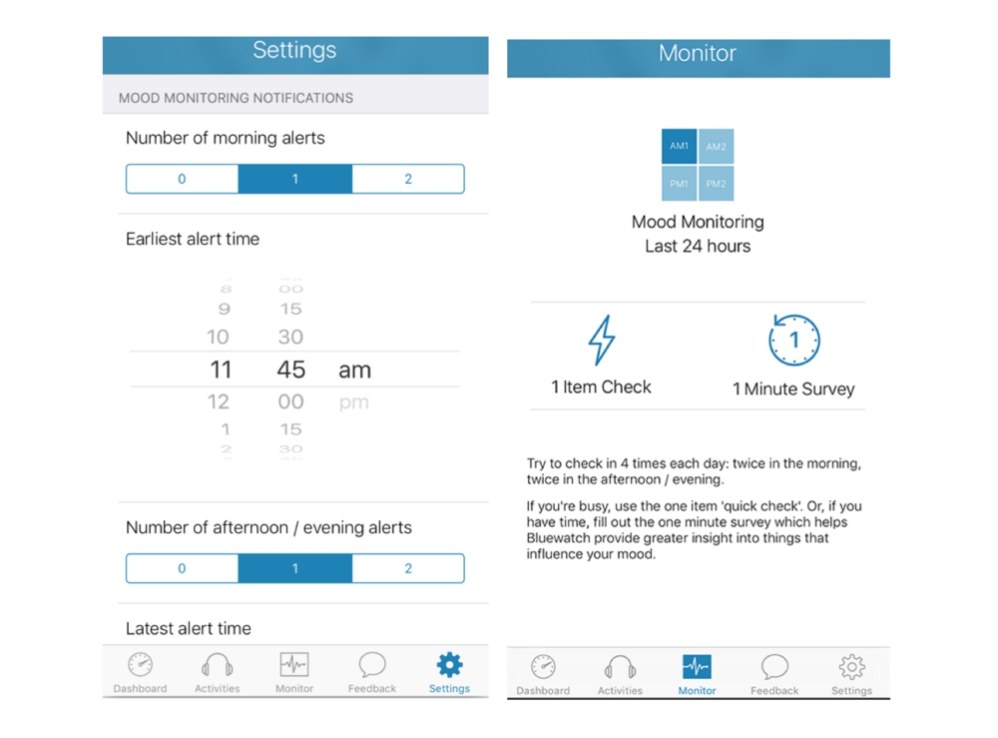
Set up of mood surveys within BlueWatch.

**Figure 4:**
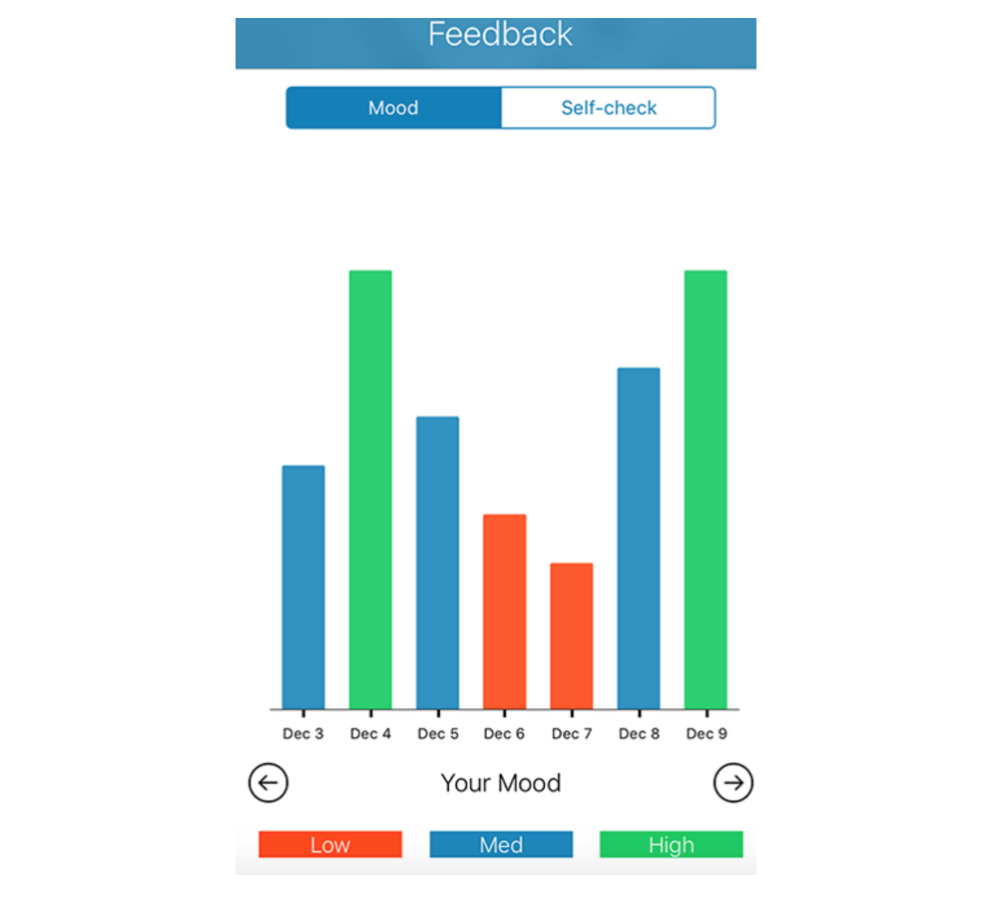
Example of a chart showing mood scores for the past week.

Given the self-guided nature of BlueWatch, several design features were added to enhance technologically mediated therapeutic alliance and engagement. The app displays a brief welcome video by default the first time a participant opens the app. This reaffirms the purpose of the intervention, instructs participants how to engage with the app, and points out key features such as push notifications, self-monitoring, and feedback on mood over time. Every time the app is opened thereafter, participants are immediately brought to their dashboard (see Figure 2), which comprises a to-do list, with the option to *favorite* any activities that are liked and may wish to be revisited. As such, the immediate tasks are easy to find and help to prevent participants from losing track of which module they are up to or how to access the relevant content. The list of recommended content is kept brief to prevent overwhelming participants with a list of things yet to be achieved. Furthermore, content is provided in the form of audio files to reduce download size, and a single calming voice is used in each audio file for continuity. Transcripts of audio files are made available (by selecting a button, rather than by default) on screen for each activity to enhance the flexibility of delivery options. Finally, a self-monitoring section is incorporated into the app to allow participants to report their mood states throughout the intervention. These brief surveys take approximately 1 to 2 min to complete and are signaled up to 4 times per day to chart the progress of the participant’s mood across the intervention phase (see Figures 3 and 4 for an example of the summary of mood data and questions from the mood survey).

#### Perceived Usability Measures

Perceived usability was evaluated using the System Usability Scale (SUS; [34]) and a qualitative interview. The SUS is an industry-standard 10-item scale that examines the perceived usability of a technological tool. Responses are measured on a 5-point Likert-type scale with 0 (*strongly disagree*) to 4 (*strongly agree*). Items are summed and then this total is multiplied by 2.5, yielding a SUS composite score between 0 and 100, with higher scores indicating higher perceptions of usability. A SUS score over 68 is considered above average. The SUS has been found as a reliable and valid tool among both experts and service users when assessing the usability of mobile apps [35].

In addition to the SUS items (which were answered in a phone interview), qualitative questions were included to probe responses to the SUS items as well as to obtain further information about what participants liked and disliked about the app. Structured questions are provided in Multimedia Appendix 1.

### Procedure

Recruitment of participants differed depending on the target group they were from. Participants in the user group were recruited after completing the 12-week intervention using BlueWatch. These end users were randomly selected and recruited by invitation via already supplied email addresses, whereas mental health clinicians and researchers were recruited by targeted email invitations that were sent to a number of metropolitan universities and clinical practice centers to recruit relevant experts in the study. Clinicians and researchers were provided with the details to download the BlueWatch app and were instructed to test the app for a period of 7 days before the phone interview.

During the semistructured interview, trained research assistants obtained participant demographics (ie, age and sex) and presented a validated usability scale (the SUS) and open-ended questions to further probe participants’ perceptions of the app’s usability. The mean interview length was 40 min (SD 9.82), and upon completion, each participant was reimbursed with an AUD $20 shopping voucher. The semistructured interviews were recorded and then transcribed.

### Data Analysis

Descriptive statistics were used for quantitative data from the SUS. Results were presented separately for the 3 groups. Both overall SUS scores and item means were reported to provide a more complete picture of perceived usability of BlueWatch. Qualitative data were organized using a coding template analysis approach [36] according to the 6 themes for evaluating the quality of eHealth apps, as proposed in Baumel et al’s [37] review of app usability studies: (1) usability, (2) visual design, (3) user engagement, (4) content, (5) therapeutic persuasiveness, and (6) therapeutic alliance. Adoption of a prespecified set of themes rather than deriving themes from interviews permitted prioritization of topics that are widely discussed in the existing literature, thus allowing more direct alignment with this literature. Furthermore, Baumel et al’s [37] framework in particular was chosen for several key reasons. First, the framework derives from an extensive review of the existing literature to identify key dimensions of perceived usability. Second, this framework has an emphasis on persuasive design elements and as such aligns with design principles underlying the creation of BlueWatch.

Initially, an additional category for miscellaneous comments was planned for qualitative analysis; however, this seventh category was not needed, given that all comments fit within these 6 initial categories. All coding was done independently by 2 researchers (LA and RO), who then discussed results to ensure consistency and agreement in coded comments. Moreover, 100% consensus was achieved for comment suitability for prespecified themes.

## Results

### Quantitative Analysis

The quantitative usability data derived from the SUS questionnaire indicated distinctions between researchers and participants. As shown in Table 1, participants tended to report higher usability and positive user experience results than those reported by researchers and clinicians. Although researchers and clinicians tended to have higher scores for items about the difficulty of use and need for support, participants in the end-user group were more likely to strongly endorse items’ ease of use and confidence using the app. Overall, the scores suggest that service users rated the app as substantially more usable than clinicians and researchers. However, it is also worth noting that there was more variability in ratings for the researcher and clinician groups, relative to the participant group.

### Qualitative Analysis

Content from the semistructured interviews is organized below according to the evaluation categories proposed by Baumel et al [37].

#### Theme 1: Usability

Service users rated the usability of the app more positively than did the expert groups, although usability comments were generally positive overall. All participants in the user group found the app easy to use and many commented that they found the way in which the activities were presented made sense and “flowed well.” This ease of use was further attributed to the welcome message that oriented the users to key features of the app, comparability of the setup of the app with other apps the participants were familiar with, and the *To Do* list as the main page to orient participants to upcoming content:

The five slides shown when initially downloading the app was enough to know what to do throughout the app.

Participant, male

If you are familiar with apps then you do not need to learn anything [technically] before using BlueWatch.

Participant, female

As soon as the activities come in, you learn by clicking on them…You do the top one first, and then you go down the list.

Participant, female

One participant from the user group also found the option to receive a text transcript of the audio files useful, particularly in situations where playing the audio was not feasible. She emphasized that this option made the app more flexible but cautioned that it took her longer to get through the content when reading and that app users with lower reading ability may not benefit from this option.

Several of the experts expressed that quite some time was required to learn and familiarize themselves with the app:

I found the navigation really tricky at the start, if I listen to two CBT sessions and it tells me to go and do an exercise I need to go back into activities. There just seemed like a lot of steps to get my head around at the start.

Clinician, female

Although the participants who were interviewed found the language easy to follow, a useful suggestion was made by a researcher to include a glossary of terms within the app in case participants are unfamiliar with or forget the meaning of key terms:

Perhaps there could be a separate tab under the activities in which you could include a glossary of key terms that way participants don’t need to go back and listen to the audio all over again.

Researcher, female

#### Theme 2: Visual Design

Both the users and experts rated the visual design of the app highly. Users appreciated the well-thought-out appearance of the app and commented that they especially liked its overall simplicity and color palette:

It was neutral and straightforward.

Participant, female

I like the blue color, I think it is an attractive app.

Researcher, female

Users and experts found the layout and presentation of content “well-organized” and “logical.” This was summed up well by a participant from the user group:

It was self-explanatory. Going through the content, there weren’t that many options…so you couldn’t get lost in it.

Participant, female

Several interviewees did, however, comment that they would prefer the text to be presented differently:

I would suggest increasing the font size—as a 37 year old I found this hard to read.

Researcher, female

When you go to the activities it can be hard to understand as the instructions and your responses are all in the same font. I would change the font or use bold to distinguish.

Participant, male

A further suggestion was to use star ratings for mood items instead of the slider scales as currently implemented (Participant, male). This participant further raised the possibility of embedding these star ratings within the activities as well or in addition to the 1-min survey section of BlueWatch.

#### Theme 3: User Engagement

There was agreement across all participants that the app was highly interactive and personalized, particularly the self-monitoring component that provided users with a graphical representation of their mood states over time and prompts to remind them to use the app (refer to Multimedia Appendix 1).

**Table 1:** Means and standard deviations for the System Usability Scale across participant, researcher, and clinician groups.

Question^a^	Participants, mean (SD)	Researchers, mean (SD)	Clinicians, mean (SD)
I think I would like to use the app frequently	2.00 (0.7)	2.00 (0.71)	2.20 (0.84)
I found the app to be unnecessarily complex	0.20 (0.44)	1.80 (1.64)	1.20 (1.09)
I thought the app was easy to use	3.40 (0.90)	3.00 (1.22)	3.00 (1.22)
I think that I would need support of a technical person to be able to use the app	0.00 (0.00)	1.40 (1.67)	1.00 (1.22)
I found the various functions in the app were well integrated	2.80 (1.01)	2.20 (0.84)	3.20 (0.45)
I thought there was too much inconsistency in the app	0.60 (0.89)	1.40 (1.14)	0.60 (0.55)
I would imagine that most people would learn to use the app very quickly	3.20 (0.45)	2.80 (1.10)	2.60 (1.14)
I found the app very cumbersome to use	0.40 (0.55)	2.80 (0.84)	2.20 (1.30)
I felt very confident using the app	3.80 (0.45)	3.00 (1.22)	2.40 (0.55)
I needed to learn a lot of things before I could get going with the app	0.40 (0.54)	1.60 (1.50)	1.20 (1.10)
System Usability Scale total score	86.00 (10.84)	60.50 (21.61)	67.00 (15.35)

^a^Responses were scored on a 5-point Likert scale ranging from 0=strongly disagree to 4=strongly agree.

One user noted:

Mood tracking was a cool aspect of the app. I treated it like my own personalized diary.

Participant, female

There was agreement from all experts that the app achieved a personalized touch as it allowed the user to adjust the app to their unique circumstance. Specifically, there was a function within the app that allowed users to change the time of the day at which alerts were sent (Figure 3), which was found to be very important for this cohort of people who may experience unusual sleeping times:

One of the important parts of this is that you can set the alerts to suit your schedule—this is so important for depression in particular because if you have someone who sleeps late they know it is unlikely they will respond to early morning prompts.

Clinician, female

Furthermore, experts commented that the graphical feedback was an important function of the app as it allowed users insight into their mood and helped them remember that they may have days on which they feel positive:

I really like the idea of being able to see your progress and that you can see how you’re going—sometimes people who are depressed think that they are constantly depressed, when they’re not, and this lets you see that that is not the case.

Clinician, female

However, several experts also noted that a lot of content was delivered via audio (although made available via text transcripts as well) and wondered whether more graphics could be incorporated to help convey the treatment information:

If a client learnt visually and understood content through graphs and animations then they would struggle to engage with this app.

Clinician, male

#### Theme 4: Content

There was agreement among all participants that the content presented was rooted in evidence. Indeed, some of the service users commented that they had been exposed to the app components in prior therapeutic exchanges, and this ensured their confidence.

All experts agreed that the content was evidence-based and many had a preference for the behavior activation module, noting its simplicity and clear delivery:

I thought the sections on behaviour activation were really well planned out and particularly that is a core treatment of depression regardless of whether you are delving into cognitions or whether you are just looking to change their behaviours. I thought it was a really nice combination of audio plus reading plus the activity.

Clinician, male

Clinicians also specifically commented that the variety of evidence-based information presented was impressive as it could cater to a wide range of preferences:

I like that you combined CBT with mindfulness—some people just don’t like CBT and that is why having mindfulness and also behavioural activation is so important.

Clinician, female

#### Theme 5: Therapeutic Persuasiveness

End-user and expert groups offered differing opinions about the suitability of the amount of content provided by the app. Several experts believed that there were too many activities within the app and that the activities themselves were too long in duration to complete:

I think it is too long the activities—it is trying to achieve so much—it is essentially replacing 12 psych session and it seems really ambitious.

Clinician, female

In contrast, service users were more accepting of the amount of content and saw this as necessary to provide different strategies to address their depressive symptoms:

I didn’t find it overwhelming, there wasn’t too much information there was the right number.

Participant, male

Furthermore, the personalization and self-monitoring aspects of the app seemed to have the desired effect:

[The self-monitoring component] made me realise about all the happy small moments that I was having so I notice that I was happier I guess and that my anxieties weren’t as big of a deal.

Participant, female

Experts agreed on the advantage of providing mood-based feedback but also raised the possibility of incorporating explicit feedback to let participants know once they had completed a module:

I wouldn’t recommend it for clients who need that feedback immediately—and I find that those with depressive symptoms do often need that to keep up their motivation.

Clinician, female

#### Theme 6: Therapeutic Alliance

However, some users believed the app provided support to an extent but lacked human contact:

The app tried to emulate a friend, but it could never actually be that friend.

Participant, female

Others felt that the app was an extra resource that they had access to whenever they needed it:

I cannot check in with my therapist every day, but I could with the app.

Participant, female

When I felt down, I remembered to use the activities presented in the app to help with the situation.

Participant, female

Most experts raised concerns that because of the lack of support offered by a one-on-one therapist, participants could not check their understanding of difficult concepts or points with someone and that this would impact their engagement:

I was concerned that there wasn’t a section in which a participant could connect with a clinician if they wanted to either solve a problem that emerged from using the app or to check understanding of particular concepts.

Clinician, male

## Discussion

### Overview

Although eHealth interventions offer a promising way to deliver psychological treatment content, dropout rates for this form of treatment administration may be higher than those for face-to-face therapy [19]. One potential contributing factor for this dropout is difficulty in the use of the app, particularly when the app is designed to be used in a self-guided fashion. In this study, we conducted a usability analysis of an eHealth intervention (BlueWatch) created using persuasive design principles to enhance user experience. Perceived usability data were obtained from end users, clinicians, and mental health researchers to evaluate potential differences in preferences and perceptions of the app.

### Principal Findings

There was broad agreement across the 3 groups (end users, health care professionals, and researchers) that the BlueWatch app had an appealing visual layout and organization of content, was engaging for users, and offered evidence-based content that ensured a range of techniques to cater to end users who may prefer different approaches for the treatment of their depressive symptoms. The expert groups (researchers and mental health professionals) particularly liked the wide range of psychological intervention strategies available in the app, feeling that this would allow individuals with different treatment preferences to each find something that might work for them. This eclectic approach is broadly in keeping with recent depression apps, such as MyCompass, that have shown benefit in offering a suite of intervention strategies [38].

A key priority in BlueWatch’s design was to leverage self-monitoring of mood as a way to teach participants about their symptoms and to retain engagement in the app. Qualitative feedback from end users suggested that this feature had the desired effect. Participants reported that the mood monitoring surveys and associated graphical feedback were a reason to return to the app and that it increased self-awareness of how their mood fluctuated over time and in relation to use of the intervention content. These findings are consistent with prior research in which use of these app-based interventions promoted increased self-awareness [39,40] and that provision of feedback had positive effects on treatment outcomes [41,42]. Participants also liked that they could adjust the timing and frequency of these mood assessments and felt that the personalized feedback helped to remind them when to use the intervention content.

Given the self-guided nature of BlueWatch, it was not surprising that all groups made mention of the lack of therapist contact. Expert groups raised concern that lack of guidance meant it was unclear whether participants were using the content appropriately. Interviews with the user group did not indicate this to be a problem as they reported that the content was straightforward, they understood how to apply the techniques offered, and found the activities easy to digest. Nevertheless, 1 participant from the user group stated that he would like therapist contact in addition to the app because he saw this contact as an opportunity to discuss actual events in his life rather than relying on exemplars from the app to guide him through daily life circumstances. Contact with the therapist would also enable immediate feedback as he discussed any problems he wished to resolve. To overcome this limitation in BlueWatch’s current capabilities, this participant suggested a more interactive element to the journaling task, such that the app might provide feedback on any journal entries to bolster a feeling of connectedness that they might receive in face-to-face therapy.

### Limitations

Several characteristics of the present sample may have impacted the results. Participants from the end-user group were invited to participate in the usability interviews if they were aged 18 to 25 years (a peak period for depression), met the diagnostic criteria for depression, and following completion of 12 weeks of use of BlueWatch. Researchers and clinicians were older and likely to have used the app for less time than the end-user group. Such differences may account for discrepancies in ratings and impressions of the app between the end-user and other 2 groups. Furthermore, participants from the end-user group were recruited after completing a 12-week intervention phase. It is possible that these individuals were more motivated and had a more positive experience of the app than those who dropped out of the study.

This study limited its evaluation to perceived usability, and hence, the obtained results are reliant on participants recognizing and conveying any issues they may have in completing app-related tasks. Although this is a common approach to usability testing [29] and the present semistructured interviews sought to evaluate key dimensions of usability, alternative approaches such as lab-based experimental studies [23,24] may offer further insights into whether participants use the app as intended. As a consequence, it remains possible that BlueWatch was positively rated by end users and yet was not used appropriately. Further evaluation across different methods of usability testing is warranted, and may consider including individuals with no prior exposure to the app as well as individuals who have used the app for some time.

### Implications

Incorporation of researcher and clinician perspectives demonstrated potential contrast in app experiences relative to end users. The greater perceived ease of use among end users may be due to greater comfort and familiarity with apps in general among younger adults. In the case of researchers, this discrepancy reinforces the need for end-user usability testing to ensure that the proposed features of one’s app have the desired effect. In so far as clinicians and researchers are a mechanism for ensuring uptake of apps, the findings of this study suggest that their recommendations might benefit from exposure to end-user feedback as well as their own perceptions of the app.

### Conclusions

Usability data from this study broadly supported the use of BlueWatch for treating depression, with particularly positive feedback received from the end-user group. Although personalization of content through self-pacing, self-monitoring of symptoms, and feedback functions may not substitute for all the benefits of in-person therapist contact, these persuasive design features of BlueWatch appear to enhance engagement with the app’s intervention content. This study is part of a larger, ongoing trial of the efficacy of BlueWatch. Results of this evaluation will further illuminate user engagement and usage patterns for those in the intervention group and whether usage rates are associated with symptom reduction postintervention.

## Appendix

Multimedia Appendix 1 Questions from semistructured interview of participants.

## Abbreviations

CBT: cognitive behavioral therapy
SUS: System Usability Scale
