# Supplementary material for: A Mobile App–Based Intervention for Depression: End-User and Expert Usability Testing Study
Source: JMIR Ment Health. 2018 Aug 23;5(3):e54. doi: 10.2196/mental.9445 (PMC6127496; doi:10.2196/mental.9445)
Supplement: Multimedia Appendix 1 [file mental_v5i3e54_app1.pdf]

Appendix B – questions from semi-structured interview of participants

Open ended questions:

1. Did you find the app to be sufficient in supporting you to reduce depressive symptoms?
2. Did you find the app to help you in real-life situations?
3. Did the app impede your ability to deal with real-life situations or real-life problems?
4. Did you feel that you became dependent on the app?
5. Did the app help you solve real-life problems?
6. Did you think the responses you gave in the app were authentic?
7. Do you think this app impedes your likelihood of seeking face-to-face interactions with mental health services?
8. Did the app discourage you from having face-to-face interactions with you family and/or friends?
9. What did you like most about the app?
10. What did you like least about the app?
11. If you could, how would you improve the app?
